# Supplementary material for: Cohesin-Mediated Genome Architecture Does Not Define DNA Replication Timing Domains
Source: Genes (Basel). 2019 Mar 4;10(3):196. doi: 10.3390/genes10030196 (PMC6471042; doi:10.3390/genes10030196)
Supplement: Supplementary file 1 [file genes-10-00196-s001.pdf]

Supplemental

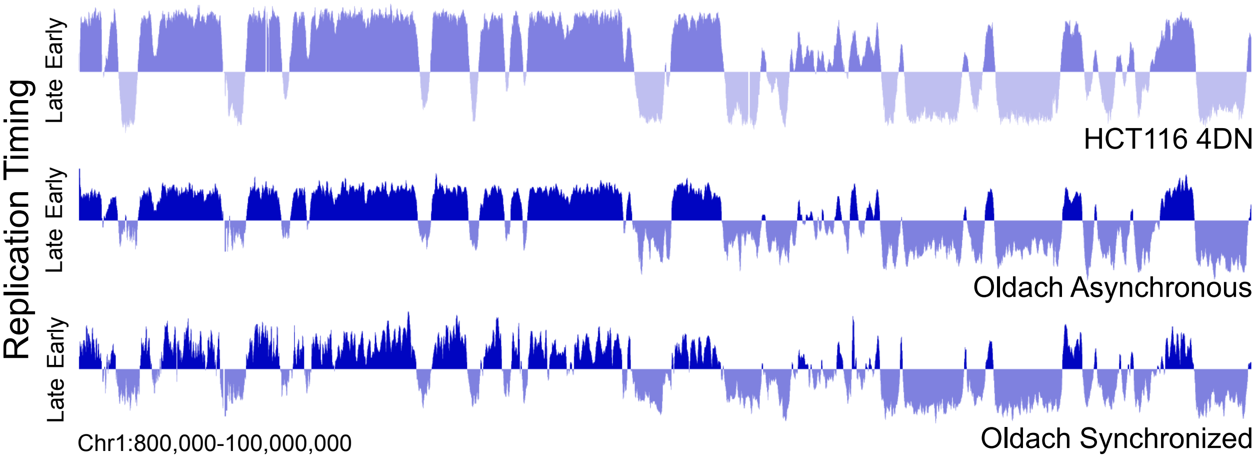

Supplemental Figure 1. Newly generated RepliSeq data agrees with previously published RepliSeq data for HCT116 cells. Example locus overlay of replication timing (RT, log-scaled ratio of read counts in early over late S phase bins) for untreated HCT116 cells from the asynchronous and synchronized experiments, compared to independent HCT116 data from the 4D Nucleome portal.

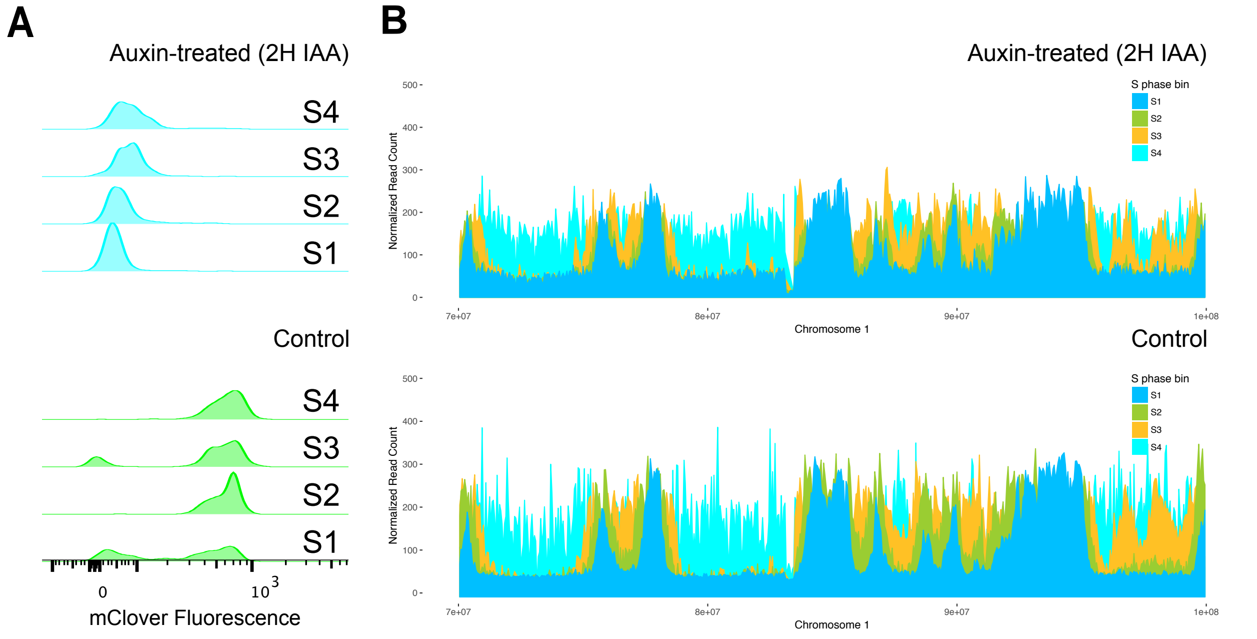

Supplemental Figure 2. Sample preparation for asynchronous RepliSeq experiment. A. Auxin-treated cells showed loss of cohesin as assayed by loss of mClover signal. B. In asynchronous sorted samples BrdU incorporation proceeds outward from specific peaks in the early S phase (S1) sample to pan-genome incorporation in the late S (S4) sample.

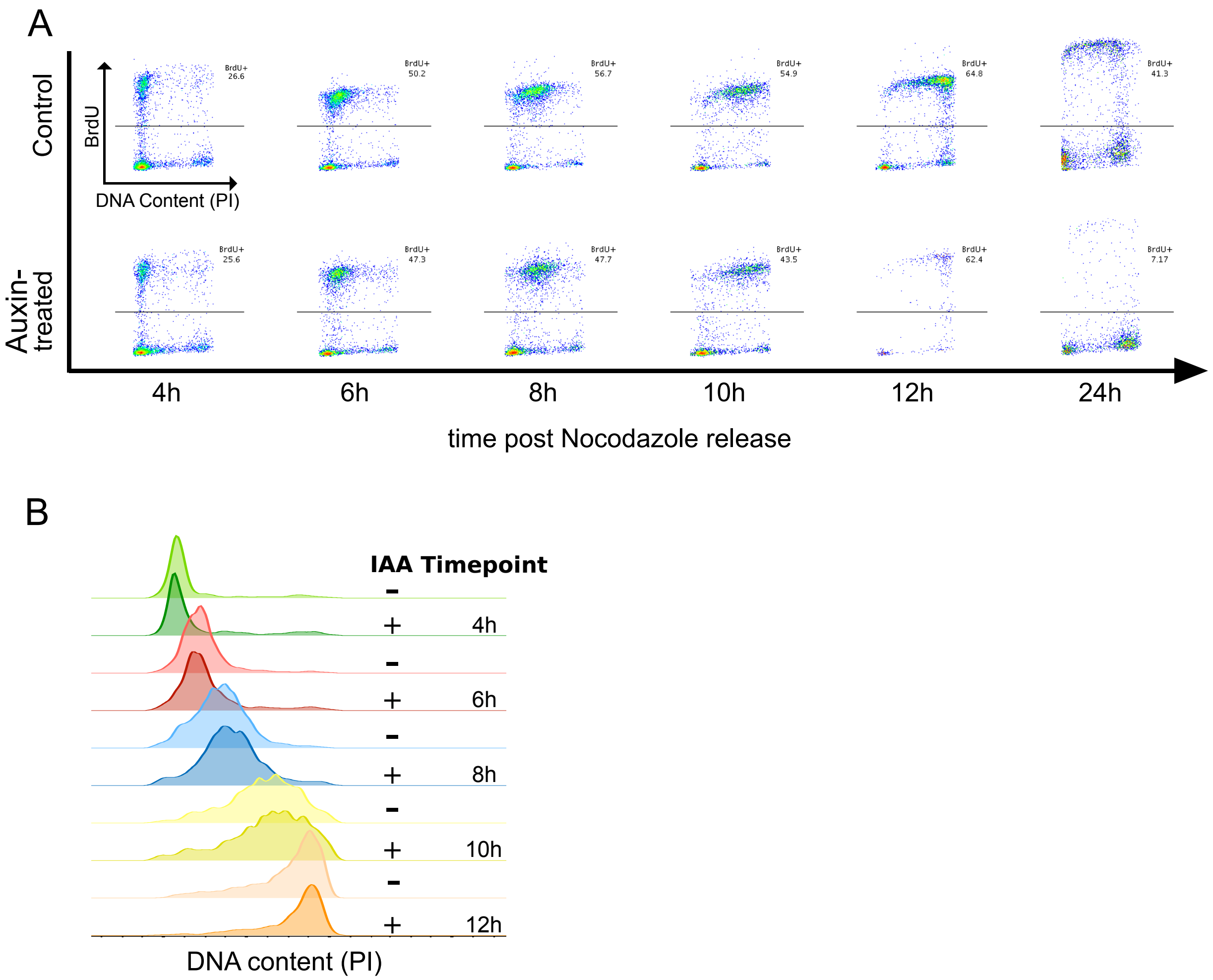

Supplemental Figure 3. Auxin treatment post release from nocodazole by mitotic shake-off does not perturb synchronous progression through S phase. A. Flow cytometry data for DNA content versus BrdU signal showing progression through S phase upon release from nocodazole by mitotic shake-off. B. DNA content as quantified by PI signal for BrdU (+) gated cell populations of auxin-treated or control samples at varying timepoints following mitotic shake-off.

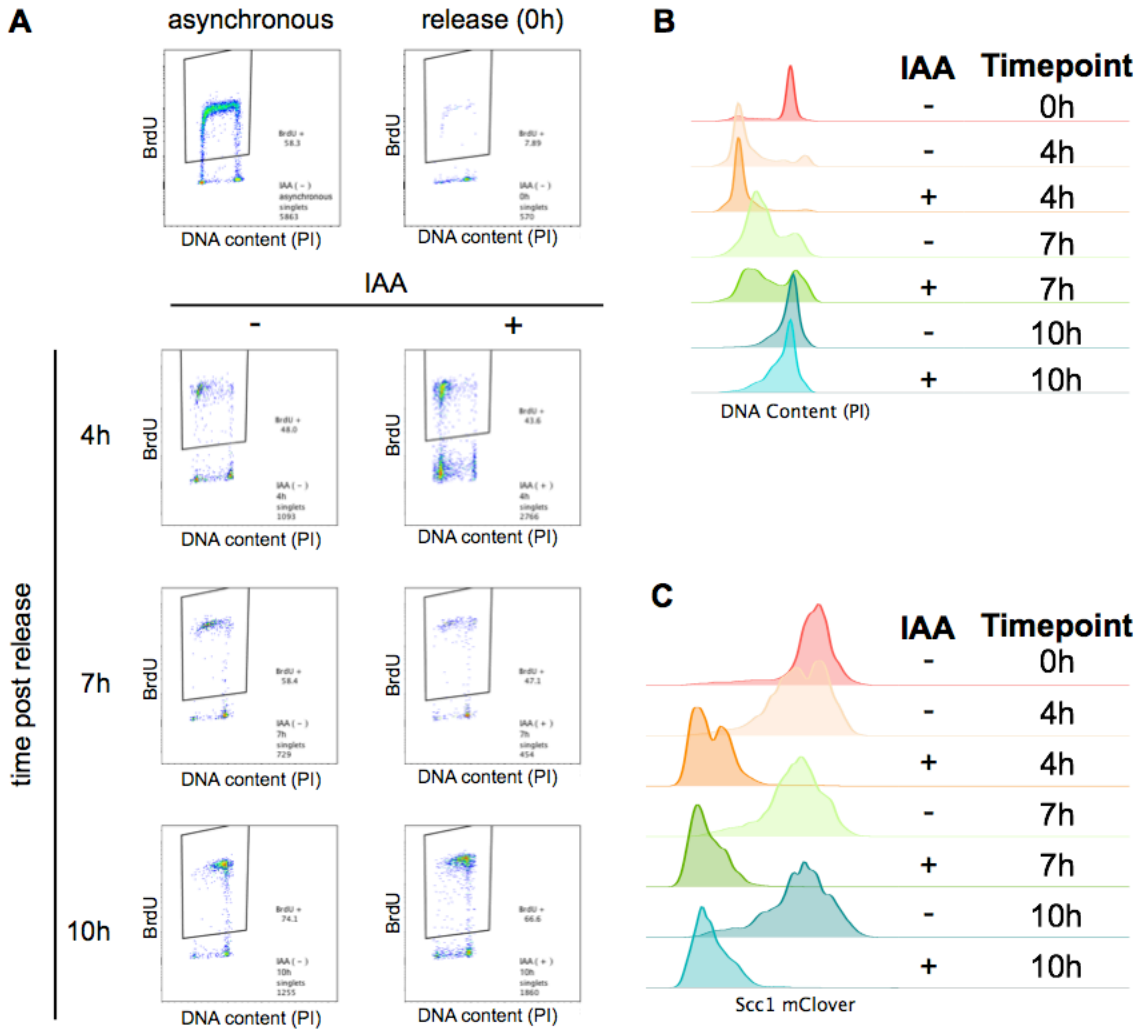

Supplemental Figure 4. Sample preparation for synchronous RepliSeq experiment A. Samples were collected at 4, 7, and 10 hours post-release to assess replication timing in early, mid, and late S phase, respectively. B. Flow profile gated on BrdU (+) singlets show synchronous progression through S phase. (0h post release sample not gated for BrdU (+) cells). C. Loss of Scc1 in synchronized samples was confirmed via loss of mClover signal.

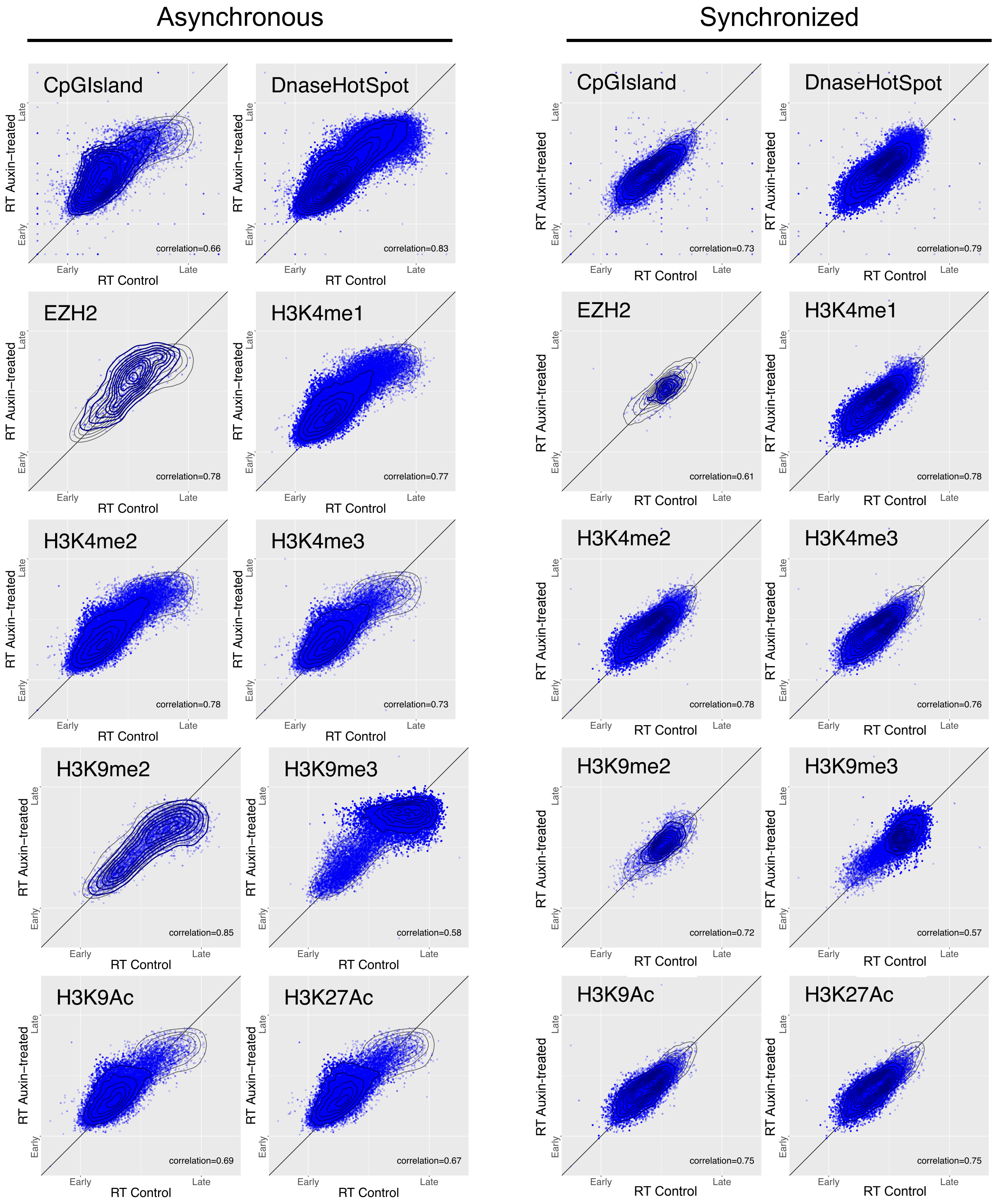

Supplemental Figure 5. Additional plots of control versus auxin-treated replication timing at genomic loci with chromatin states or protein binding sites associated with characteristically early or late replication timing.

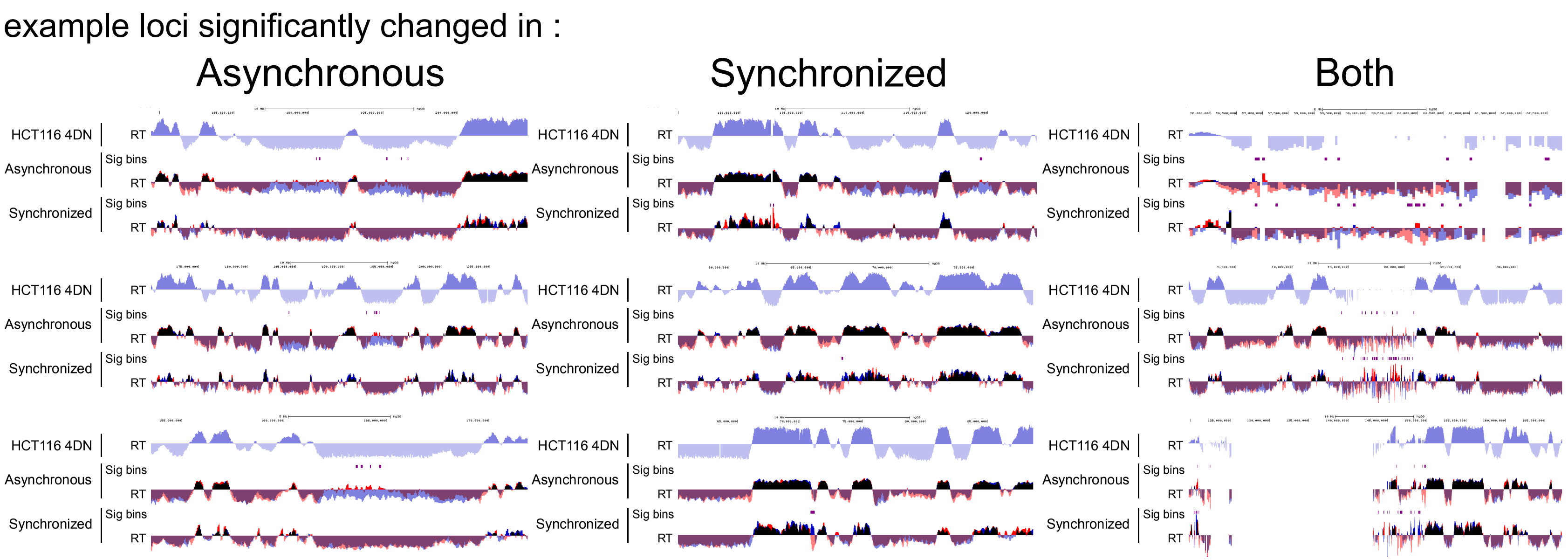

Supplemental Figure 6. Loci with significant changes to replication timing, in asynchronous, synchronized, or both experiments. Significance determined by FDR adjusted p value < 0.05.

Supplemental Table 1. Source for genomic features annotated in HCT116 cells.

Feature	Source
Super enhancers	(Hnisz *et al*, 2013) [45]
H3K9me3	ENCSR179BUC
H3K9me2	ENCSR555LYM
CTCF	ENCSR000BSE
H3K27Ac	ENCSR000EUT
H3K9Ac	ENCSR093SHE
H3K4me3	ENCSR333OPW
H3K4me2	ENCSR794ULT
H3K4me1	ENCSR161MXP
H4K20me1	ENCSR474DOV
H2AFZ	ENCSR227XNT
EZH2	ENCSR046HGP
Dnase Hotspot	wgEncodeRegDnaseUwHCT116Peak (Sabo *et al*, 2006) [46] via UCSC Table Browser
CpG Island	cpgIslandExt via UCSC Table Browser
